# Disease modifying and antiangiogenic activity of 2-Methoxyestradiol in a murine model of rheumatoid arthritis

**DOI:** 10.1186/1471-2474-10-46

**Published:** 2009-05-01

**Authors:** Stacy M Plum, Eun J Park, Steve J Strawn, Elizabeth G Moore, Carolyn F Sidor, William E Fogler

**Affiliations:** 1 EntreMed, Inc. 9640 Medical Center Drive, Rockville, MD 20850, USA

## Abstract

**Background:**

A critical component of disease progression in rheumatoid arthritis (RA) involves neovascularization associated with pannus formation. 2-methoxyestradiol (2ME2) is a naturally occurring molecule with no known physiologic function, although at pharmacologic concentrations it has antiproliferative and antiangiogenic activities. We investigated the impact of orally administered 2ME2 on the initiation and development of proliferative synovitis using the anti-collagen monoclonal antibodies (CAIA) model.

**Methods:**

Severe polyarticular arthritis was induced in Balb/c female mice by administration of 2 mg of a monoclonal antibody cocktail intravenously into the tail vein of mice. Twenty-four hours following monoclonal antibody administration, mice were injected with 25 μg of LPS (*E. coli *strain 0111:B4) via the intraperitoneal route. Treatment with 2ME2 (100, 75, 50, 25, 10, 1 mg/kg, p.o., daily), or vehicle control began 24 hrs following LPS challenge and continued to day 21. Hind limbs were harvested, sectioned and evaluated for DMARD activity and general histopathology by histomorphometric analysis and immunohistochemistry (vWF staining). In a separate study, different dosing regimens of 2ME2 (100 mg/kg; q.d. *vs *q.w. *vs *q.w. × 2) were evaluated. The effect of treatment with 2ME2 on gene expression of inflammatory cytokines and angiogenic growth factors in the joint space was evaluated 5 and 14 days after the induction of arthritis.

**Results:**

Mice treated with 2ME2 beginning 24 hours post anti-collagen monoclonal antibody injection, showed a dose-dependent inhibition in mean arthritic scores. At study termination (day 21), blinded histomorphometric assessments of sectioned hind limbs demonstrated decreases in synovial inflammation, articular cartilage degradation, pannus formation, osteoclast activity and bone resorption. At the maximal efficacious dosing regimen (100 mg/kg/day), administration of 2ME2 resulted in total inhibition of the study parameters and prevented neovascularization into the joint. Examination of gene expression on dissected hind limbs from mice treated for 5 or 14 days with 2ME2 showed inhibition of inflammatory cytokine message for IL-1β, TNF-α, IL-6 and IL-17, as well as the angiogenic cytokines, VEGF and FGF-2.

**Conclusion:**

These data demonstrate that in the CAIA mouse model of RA, 2ME2 has disease modifying activity that is at least partially attributable to the inhibition of neovascular development. Further, the data suggests new mechanistic points of intervention for 2ME2 in RA, specifically inhibition of inflammatory mediators and osteoclast activity.

## Background

Rheumatoid arthritis (RA) is a chronic inflammatory disease that is characterized by progressive joint damage. The pathology of RA is complex and mediated by several mechanisms. Early stages of disease progression are defined by capillary formation, hyperplasia of the synovial membrane, influx of leukocytes and inflammatory cells, and hypertrophic synoviocytes. Established RA exhibits cellular infiltration, pannus formation, cartilage degradation, bone erosion and extensive angiogenesis restricted to the synovium [1,2].

Improved understanding of the molecular mechanisms supporting the pathogenesis of rheumatoid arthritis has revealed new targets for therapeutic intervention. One such novel target for disease modulation is rheumatoid arthritis-associated angiogenesis [3,4]. Specifically, in the context of RA, angiogenesis plays a critical role in perpetuating inflammatory and immune responses, as well as supporting pannus growth and development.

2-Methoxyestradiol (2ME2) is an endogenous, naturally-occurring metabolite of estradiol with a low affinity for the estrogen receptor (0.05%). It has antiproliferative, antiangiogenic and proapoptotic activity [5,6]. Mechanistically, 2ME2 binds to the colchicine binding site of tubulin causing microtubule depolymerization and the down-regulation of transcription factors, hypoxia inducible factor 1-alpha (HIF1-α), NF-κB, and Stat-3 [7-10]. 2ME2 inhibits tumor-associated angiogenesis and malignant progression in multiple tumor models in the absence of dose-limiting toxicities. Phase 1 & 2 clinical trials in oncology have been conducted with an oral formulation of 2ME2 (Panzem^® ^NCD) and manageable changes in liver function tests and hypophosphatemia have been described in some patients.

The antiarthritic activity of 2ME2 in preclinical models of RA has been previously described [11-13]. In two of these studies the potential impact of 2ME2 on angiogenesis was directly assessed and conflicting data was generated. 2ME2 failed to block synovial angiogenesis in sections stained with laminin in a rat adjuvant-induced arthritis model. In contrast, 2ME2 was shown to block articular angiogenesis in a rat collagen-induced arthritis model as measured by vWF staining and decreased synovial gene expression of vascular endothelial growth factor and fibroblast growth factor.

In the present study, we determined the impact of 2ME2 in a mouse CAIA model. While special emphasis was placed on ascertaining the relationship between 2ME2-induced antiangiogenic and antiarthritic activity, novel information was also obtained concerning the effects of 2ME2 on additional indicators of disease attenuation. The results show that 2ME2 has disease-modifying activity that is at least partly attributable to the inhibition of neovasculature development. In addition, 2ME2 impacts additional mechanisms involved in the progression of joint disease, specifically inhibition of inflammation and bone resorption.

## Methods

### Animals and Therapeutic Agents

Specific pathogen free 5 to 7 week old Balb/C female mice were purchased from the Jackson Laboratory (Bar Harbor, ME) and housed in a barrier facility. In conducting the research in this report the investigators adhered to the Principles of Laboratory Animal *Care (NIH Publication No. 85-23)*. 2ME2 in a NanoCrystal^® ^colloidal dispersion (NCD) was manufactured by Elan Drug Delivery (King of Prussia, PA). Control diluent used in these studies comprised the NCD without 2ME2. In conducting the research in this report, the investigators adhered to the Guide for Laboratory Animals and Care of the Institute of Laboratory Animal Resources, National Academy of Sciences, National Research Council.

### Induction of CAIA by Anti-Collagen Monoclonal Antibodies and LPS

CAIA was induced by the intravenous injection of monoclonal antibodies to CII (Arthrogen-CIA, Chemicon International, Inc., Temcula, CA), according to the manufacturers procedure. Arthrogen-CIA is an arthritis-inducing mixture of four monoclonal antibodies to anti-CII resuspended in sterile Dulbecco's PBS. All four monoclonal antibodies in this mixture recognize the conserved epitopes (CB11) shared by various species of CII and cross-react with homologous and heterologous CII. Three of these four monoclonal antibodies (F10-21, A2-10, and D8-6) recognize epitopes clustered within an 84 amino acid residue fragment. LyC2, mof CB11, and the fourth monoclonal antibody (D1-2G) react with LyC1. Arthritis is mediated in this model by immune complex mediated complement activation [14]. Two mg of Arthrogen-CIA per mouse were injected intravenously into the tail vein of mice. Twenty-four hours following monoclonal antibody administration, mice were injected with 25 μg of LPS (*E. coli *strain 0111:B4) via the intraperitoneal route. Treatment with 2ME2 (100, 75, 50, 25, 10, 1 mg/kg, p.o., daily), or vehicle control (began 24 hrs following LPS challenge and continued to day 21. In a separate study, different dosing regimens of 2ME2 (100 mg/kg; q.d. *vs *q.w. *vs *q.w. × 2) were evaluated. The extent of disease was scored in a blinded fashion by visual observation using the following scale: 0 – no signs of involvement, 1 – redness, red spots on paw, 2 – partial swelling, difficulties with stretching paw; 3 – swelling, no loading on paw; 4 – maximally swollen paw, no loading. The clinical arthritis score is the sum of arthritic scores from 4 footpads. Maximal arthritic score is 16 for each mouse. At necropsy, both right and left hind paws were collected, preserved in 10% buffered formalin, stored at 4°C and processed for histopathology, histomorphometric analyses and immunohistochemistry (Skeletech, Inc. Bothell, WA).

### Qualitative Histopathology

Paws were decalcified with 5% formic acid, dehydrated through a series of ascending ethanol solutions and embedded in paraffin. Saggital sections, through the middle of the hind paw, were obtained using a rotary microtome (Model RM 2165, Leica Microsystems Inc., Bannockburn, IL). Sections of 4 μm thickness were stained with toluidine blue and further developed for tartrate-resistant acid phosphatase reactivity. Alternatively, sections were stained for von Willebrand factor (vWf) expression to determine vascularity. All histopathology scoring was performed blinded. The area of evaluation included the talus-navicular joint through the navicular to the second metatarsal joint. The scoring criteria were as follows:

#### Cell Infiltration Score

Grade 1: Normal; Grade 2: Mild, subsynovial cell fibrosis with mild cellular infiltrate consisting primarily of mononuclear type cellular infiltrate. Grade 3: Moderate, subsynovial cell fibrosis and mononuclear type cellular infiltrate. Grade 4: Marked, periarticular inflammatory infiltrate, periarticular fibrosis and marginal osteophyte formation.

#### Pannus Severity Score

Grade 1: Normal; Grade 2: Mild, vascularized connective tissue at the margins of the joint; Grade 3: Moderate, vascularized connective tissue involving up to 50% of the cartilage surface; Grade 4: Marked, vascularized connective tissue covers more than 50% of the cartilage surface.

#### Cartilage Lesion Severity Score

Grade 1: Normal; Grade 2: Mild loss of matrix staining and articular cartilage surface damage; Grade 3: Moderate, clear reduction of the matrix staining and damage to the articular cartilage. Grade 4: Marked, ankylosis of the joint with marked loss of the articular cartilage.

#### Bone Resorption Severity Score

Grade 1: Normal; Grade 2: Mild appearance of bone resorption at the margin of the bone. Grade 3: Moderate, bone resorption involving subchondral bone with intact articular cartilage and bone interface: Grade 4: Marked, bone resorption involving extensive subchondral and marginal area with loss of intact articular cartilage and bone interface.

### Quantitative Histomorphometric Analysis

Disease manifestation in the stained sections was quantified using Osteomeasure software (Osteometer, Atlanta, GA). The following five parameters were either obtained directly by tracing and dotting or were calculated: percentage of damaged articular surface, percentage of articular area without proteoglycan staining, thickness of articular cartilage, thickness of articular cartilage without proteoglycan staining and osteoclast number per marrow area.

### Cytokine Analysis

Hind limbs, including the ankle joint were removed and snap frozen in liquid nitrogen. TRIzol, (1 mL; Invitrogen, Carlsbad, CA) was added to the joint, which was subsequently homogenized using a polytron homogenizer (PT1300D, Kinematica AG, Newark, NJ). Homogenate was transferred to Eppendorf tubes and spun for 10 min at 1000 rpm. Supernatants were removed and 200 μl of chloroform was added to 1 mL of supernatant, which was then spun for 15 min at 12,000 rpm at 4°C. The aqueous layer was removed and an equal volume of ethanol was added for RNA extraction. Extraction of total RNA from mouse hind limbs was performed using the RNeasy mini kit (Qiagen, Germantown, MD) as per the manufacturer's instructions. Quantity of RNA and its purity was measured by reading absorbance at 260 and 280 nm. One μg of total RNA was used to prepare cDNA using Taqman Reverse Transcription reagents (Applied Biosystems, Warrington, England) as per the manufacturer's instructions. Real time PCR was performed using the ABI7300 Sequence Detection systems (Applied Biosystems, Warrington, England). Pre-designed Taqman Gene Expression Assays (Applied Biosystems) for IL-6 (assay ID Mm00446190_m1), IL-1β (assay ID Mm00434228_m1), TNF-α (assay ID Mm00443258_m1), VEGF (assay ID Mm00437304_m1) FGF-2 (assay ID Mm00433287_m1), and IL-17 (assay ID Mm00433287_m1) were used to detect gene expression. Taqman Eukaryotic 18s rRNA was used as an endogenous control. The PCR conditions were as follows: an initial incubation at 50°C for 2 min, then a denaturation at 95°C for 10 min followed by 50 cycles of 95°C for 15 sec and 60°C for 1 min. The relative quantitation of gene expression was performed using the comparative CT (DDCT) method.

### Statistical Analysis

Statistical analyses were determined by non-parametric Wilcoxon test for comparisons of arthritic scores and real time PCR quantitation. The significance of the arthritic score was determined on study day 10 post-arthritis induction. For histomorphometric analysis, data on study day 21 from right and left hind paws from individual animals were combined for the purpose of analysis. Group summaries were presented as mean and standard deviation. Qualitative histopathology parameters were statistically analyzed using a non-parametric Wilcoxon test. Qualitative histomorphometry parameters were analyzed using an unpaired t-test. Each group was individually compared to all other treated groups. The statistical analyses were conducted using SAS statistical analysis software (SAS Institute, Cary, NC).

## Results

### 2ME2 Treatment Dose-Dependently Reduces Arthritic Score in a Mouse Model of Rheumatoid Arthritis

**Figure 1:**
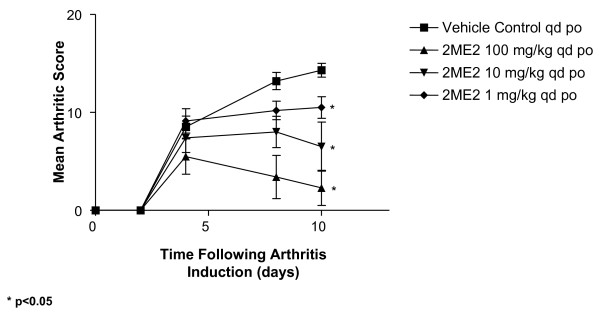
**Daily Gavage with 2ME2 Decreases Arthritic Score in an Antibody-Induced Model of Rheumatoid Arthritis**. Female Balb/c mice (5–7 weeks of age) were injected i.v. with a monoclonal antibody cocktail against type II collagen (2 mg/mouse). Twenty-four hours later, mice were treated i.p. with 25 μg LPS (E. coli strain 0111:B4). Subsequent to LPS administration, cohorts of mice (n = 15/group) began treatment by daily oral gavage with either vehicle control or 2ME2 at 1, 10, or 100 mg/kg for 21 consecutive days. Mice were assessed on study days 2, 4, 8, and 10 for clinical severity of disease progression by observing paw swelling, erythema, and a loss of passive use. Maximum arthritic score is 16 for each mouse. Asterisks indicate groups statistically different from vehicle control on study day 10 (p < 0.05). This experiment is representative of 4 experiments.

**Figure 2:**
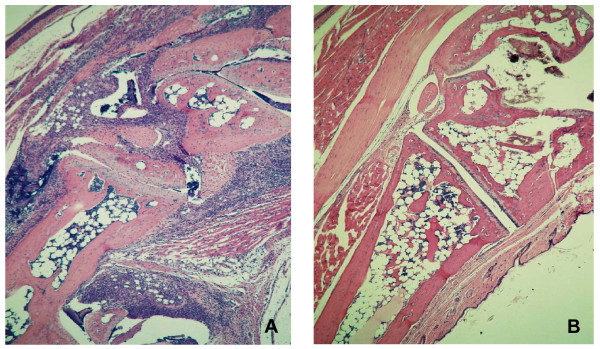
**Abrogation of the Articular Inflammatory Response in Mice Treated with 2ME2**. On study day 8, right and left hind paws were fixed in formalin, sectioned and stained by H&E. Intense inflammation is evident in the joints of mice receiving treatment with control diluent (A). In contrast, treatment of mice with 100 mg/kg/day of 2ME2 has essentially abrogated the inflammatory response (B). Magnification = 10×.

To investigate the antiarthritic effect of 2ME2, the collagen antibody-induced arthritis model in mice was employed. In this model, arthritis is induced in Balb/c mice by the combination of a sub-arthrogenic dose of four monoclonal antibodies to type II collagen followed by lipopolysaccharide (LPS). Oral administration with control diluent or 2ME2 (1, 10, and 100 mg/kg, q.d.) was initiated 2 h following LPS administration and continued for 21 consecutive days. Mice were assessed on study days 2, 4, 8, and 10 for disease progression by assessing clinical severity of paw swelling, erythema, and loss of passive use from all four footpads. Robust joint inflammation developed in animals receiving vehicle control, with a maximal mean clinical score of 14.2 reached by study day 10 (Figure 1 and 2). Mice treated by daily oral gavage with 2ME2 had significantly reduced clinical scores on study day 10 and the extent of joint involvement was dose-dependent. As compared to control-treated mice, 2ME2 doses of 1, 10, and 100 mg/kg/day inhibited arthritic score by 26, 54, and 83%, respectively (Figure 1, p < 0.05 for all three doses when compared to vehicle control). In an additional study, the antiarthritic activity of 2ME2 was further evaluated by increasing the doses assessed to 100, 75, 50, 25 10 mg/kg, p.o. daily. Under these experimental conditions, the maximum antiarthritic effect was achieved with oral administration of 75 or 100 mg/kg daily (data not shown).

**Figure 3:**
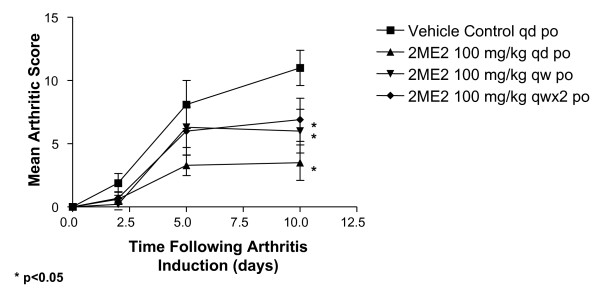
**Maximum Antiarthritic Activity is Observed With Daily Dosing with 2ME2**. Female Balb/c mice (5–7 weeks of age) were injected i.v. with a monoclonal antibody cocktail against type II collagen (2 mg/mouse). Twenty-four hours later, mice were treated i.p. with 25 μg LPS (E. coli strain 0111:B4). Subsequent to LPS administration, cohorts of mice (n = 10/group) began treatment by daily oral gavage with either vehicle control or 2ME2 at 100 mg/kg for 10 consecutive days. Additional cohorts of mice were treated weekly or twice a week with 2ME2 at 100 mg/kg. Mice were assessed on study days 2, 4, 8, and 10 for clinical severity of disease progression by observing paw swelling, erythema, and a loss of passive use. Maximum arthritic score is 16 for each mouse. Asterisks indicate groups statistically different from vehicle control on study day 10 (p < 0.05).

To evaluate whether daily dosing was required to elicit antiarthritic activity, a dose regimen evaluation was performed in the same model. Robust joint inflammation developed in animals receiving vehicle control, with a maximal mean clinical score of 11.1 reached by study day 10 (Figure 3). Daily dosing of 2ME2 (100 mg/kg) resulted in 68% inhibition of arthritis in the joints. Reducing administration of 2ME2 (100 mg/kg) to twice a week or once weekly regimen resulted in a decrease in antiarthritic activity (45 and 37% inhibition, respectively). While all three dosing regimens resulted in statistically significant differences when compared to vehicle control treated animals (P < 0.05), daily dosing with 2ME2 generated antiarthritic activity that was statistically different from both weekly and twice weekly treatment. The weekly regimens were not statistically different from each other.

### Treatment with 2ME2 Inhibits Parameters of Disease Progression

**Figure 4:**
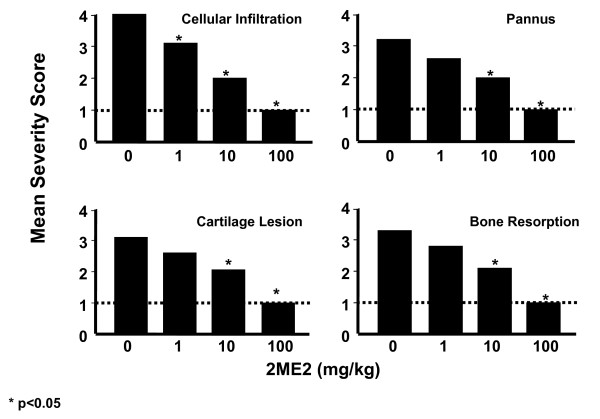
**Effect of 2ME2 on Qualitative Histopathology Parameters**. Five mice from each group were necropsied on study day 21. At necropsy, both right and left hind paws were collected, preserved in 10% buffered formalin, and processed for histopathology and histomorphometric analyses. Qualitative histopathology scoring was performed using acid phosphatase/toluidine blue stained sections on blinded samples. The area of interest included the area from the talus-navicular joint to the navicular to the second metatarsal joint. Asterisks indicate groups statistically different from vehicle control (p < 0.05).

To determine the impact of 2ME2 treatment on joint inflammation and destruction, mice were necropsied on study day 21 and both right and left hind paws were processed for histopathology and histomorphometric analyses (Skeletech, Bothell, WA). The tissue sections were blinded and graded for severity of cellular infiltration, pannus formation, cartilage lesions and bone resorption using a 1–4 scaling metric. At study termination (day 21), the articular joints from mice treated with vehicle control showed a pronounced cellular infiltration, pannus, and erosions in both cartilage and bone (Figure 4). Under identical experimental conditions, mice treated with 2ME2 demonstrated a dose-dependent attenuation of leukocyte infiltration, pannus severity, and destruction of cartilage bone (Figure 4). As compared to vehicle control-treated mice, the lack of development in all four parameters of disease progression following 2ME2 treatment at 10 or 100 mg/kg/day was statistically significant. Notably, treatment with 100 mg/kg/day of 2ME2 was nearly 100% effective in the inhibition of the inflammatory response and tissue destructive processes in this model.

**Figure 5:**
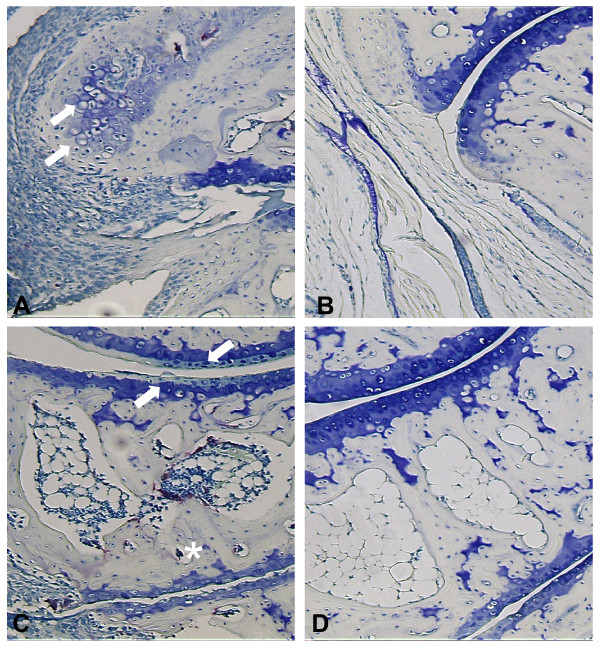
**Inhibition of Disease Progression by Treatment with 2ME2**. On study day 21, periarticular inflammatory infiltrates (arrow heads), fibrosis (asterisks) and osteophyte (arrows) are readily apparent in mouse tissue following induction with monoclonal antibodies to type II collagen and treatment with vehicle control (A). In contrast, treatment of mice with 100 mg/kg/day 2ME2 resulted in abrogation of these histopathologic changes and the appearance of normal architecture (B). Loss of articular proteoglycan (arrows) and induction of osteoclast activity (asterisk) were observed in tissue from mice treated with vehicle control (C). In contrast, treatment of mice with 100 mg/kg/day 2ME2 resulted in abrogation of these histopathologic changes and the appearance of normal joint architecture (D). Magnification = 60×.

When the joint sections were evaluated histologically, periarticular inflammatory infiltrates, fibrosis and osteophyte formation were readily apparent in mouse tissue following treatment with vehicle control (Figure 5A). These pathological phenomenon were dose-dependently inhibited with 2ME2 treatment. At the highest dose of 2ME2 (100 mg/kg/day) infiltration of inflammatory cells and osteophyte formation were completely blocked (Figure 5B). In fact, histology from joints treated with this dose of 2ME2 was indistinguishable from normal non-arthritic joints.

In addition to scoring the qualitative parameters described above, quantitative histomorphometric analysis was performed on the sectioned paws. Assessment of total osteoclast number per marrow area revealed increased osteoclastic activity in the joints of mice treated with vehicle control (34 osteoclasts in the vehicle control group). A dose-dependent decrease in osteoclasts was evident in animals treated with 2ME2 (Table 1). In fact, treatment with 100 mg/kg/day of 2ME2 resulted in complete ablation of osteoclast numbers (zero osteoclasts present). In TRAP stained sections, loss of articular proteoglycan and induction of osteoclast activity were readily apparent in mouse tissue from animals treated with vehicle control (Figure 5C). In contrast, treatment of mice with 100 mg/kg/day 2ME2 resulted in abrogation of these histopathologic changes and the appearance of normal joint architecture (Figure 5D). Treatment with this dose of 2ME2 was able to salvage potentially damaged articular cartilage resulting in a reduction in damaged articular cartilage surface and areas of articular cartilage that lack proteoglycan staining and an increase in thickness of total articular cartilage area to normal appearance.

**Table 1:** Effect of 2ME2 on Quantitative Histomorphometry Parameters

**Treatment Group**	**Total Osteoclast Numbers**	**Articular cartilage area without proteoglycan staining (%)**	**Damaged articular cartilage surface (%)**	**Thickness of total articular cartilage area (μM)**
Vehicle Control	33.9	17.23	47.3	26.06
2ME2 100 mg/kg	0	0.18*	0*	32.64*
2ME2 10 mg/kg	5.7*	6.71	27.9	27.34
2ME2 1 mg/kg	21.9*	11.21	37.0	27.5

Quantitative histomorphometry was performed using Ostemeasure software interfaced with a Nikon Eclipse E400 light/epiflourescent microscope and video system. Histomorphometric measurements of the articular area and surface were performed using Safranin O stained slides and osteoclast number was counted using acid phosphatase/toluidine blue stained slides using blinded samples. The area of interest included the area from the talus-navicular joint to the navicular to the second metatarsal joint. Results are presented as mean determinations from 10 observations (*p < 0.05 compared to vehicle control).

### 2ME2 Completely Blocks Neovascularization Associated with CAIA

**Figure 6:**
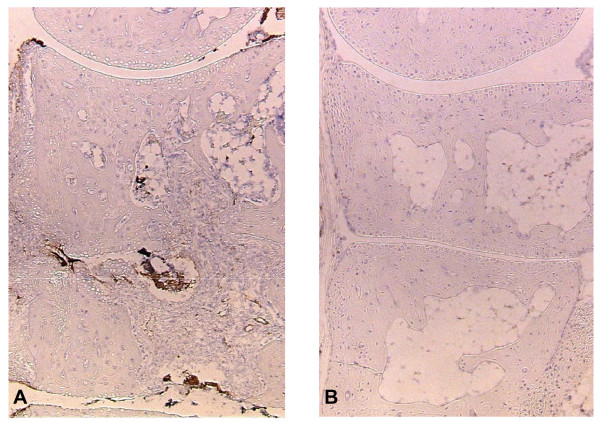
**2ME2 Inhibits Angiogenesis in the Antibody-Induced Model of Rheumatoid Arthritis**. On study day 21, right and left hind paws were fixed in formalin, sectioned and stained for vWF, a marker of angiogenesis and endothelial cell proliferation. Abundant staining for vWF factor was evident in sections from mice treated with vehicle control (A). In contrast, treatment of mice with 100 mg/kg/day of 2ME2 completely blocked vWF staining (B). Magnification = 10×.

2ME2 is well characterized as an antiangiogenic agent in a number of oncology models. Angiogenesis is known to play a prominent role in pannus formation in rheumatoid arthritis. To assess a potential mechanism by which 2ME2 may be exerting its antiarthritic activity, we stained joint samples for von Willebrand factor (vWF), a marker used to visualize angiogenesis. Hind limbs from animals treated with the control diluent or 2ME2 (100 mg/kg/day) were collected on Day 21, fixed in 10% buffered formalin, sectioned and stained. While a significant amount of vWF staining was present in sections from mice treated with vehicle control (Figure 6A), there was complete abrogation of angiogenesis as measured by vWF staining in sections from mice treated with 100 mg/kg/day of 2ME2 (Figure 6B).

### 2ME2 Blocks Gene Expression of Angiogenic and Inflammatory Cytokines in Arthritic Limbs

**Figure 7:**
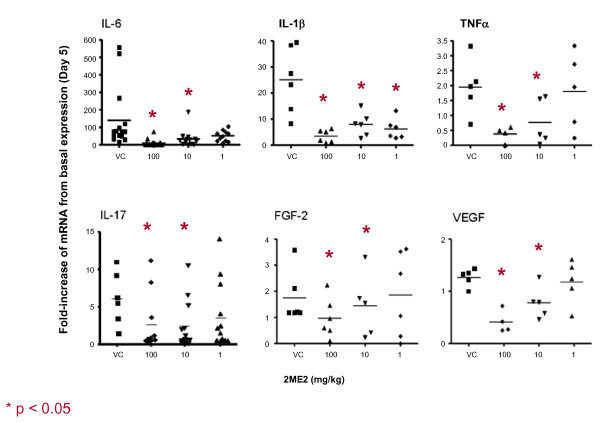
**Effect of 2ME2 on Expression of Angiogenic and Inflammatory Cytokines**. Total RNA from mouse hind limbs on study day 5 was isolated and gene expression of inflammatory (IL-6, IL-1b, TNF-a, IL-17) and angiogenic cytokines (VEGF, FGF-2) was assessed. Expression of all molecules evaluated were down-modulated upon treatment with 2ME2.

**Figure 8:**
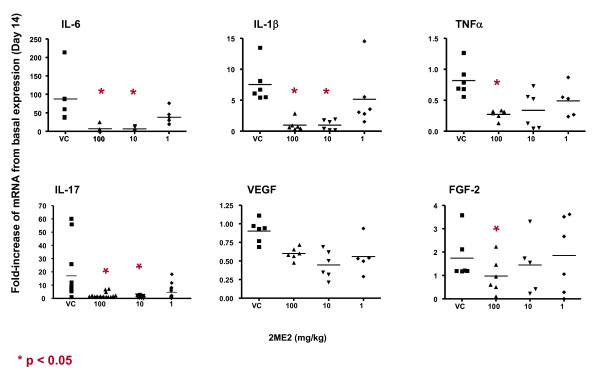
**Effect of 2ME2 on Expression of Angiogenic and Inflammatory Cytokines**. Total RNA from mouse hind limbs on study day 14 was isolated and gene expression of inflammatory (IL-6, IL-1b, TNF-a, IL-17) and angiogenic cytokines (VEGF, FGF-2) was assessed. Expression of all molecules evaluated were down-modulated upon treatment with 2ME2.

To further investigate the mechanism of the antiangiogenic and anti-inflammatory effects of 2ME2 in this animal model of rheumatoid arthritis, gene expression of angiogenic factors and inflammatory molecules in afflicted joints were evaluated. Hind limbs from mice treated with either vehicle control or 2ME2 were harvested and homogenized on study days 5 and 14. mRNA was extracted from these samples and gene expression for the pro-angiogenic molecules, vascular endothelial growth factor (VEGF) and basic fibroblast growth factor (FGF-2), and gene expression for the pro-inflammatory molecules TNFα, IL-1β, IL-6 and IL-17 were established at both time points. Mice treated daily with 2ME2, demonstrated suppressed expression levels of angiogenic (VEGF, FGF-2) and inflammatory (TNFα, IL-1β, IL-6 and IL-17) molecules on study day 5 (Figure 7). Expression levels remained suppressed when assessed on study day 14 (Figure 8).

## Discussion

Data presented in this manuscript demonstrates that the administration of 2ME2 in a mouse collagen antibody-induced model of rheumatoid arthritis leads to marked inhibition of arthritic disease. We show that 2ME2 modifies disease progression through (i) inhibition of articular angiogenesis, (ii) attenuation of inflammatory cell infiltration into the synovial compartment, (iii) abrogation of soft tissue and bone lesions, and (iv) arrest of pannus development. In addition, a number of cytokines important for recruitment of inflammatory cells and bone remodeling were modulated in the diseased joint area by daily oral administration of 2ME2. These data, taken together, support a potential role for 2ME2 as a disease modifying agent for rheumatoid arthritis. The mechanism(s) by which 2ME2 exerts its preclinical antiarthritic activity likely involves multiple pathophysiological processes.

New blood vessel formation is one of the many processes required for the pathogenesis of proliferative synovitis, of which RA is the prototype [15-18]. Studies have demonstrated that multiple proangiogenic molecules are present in the rheumatoid synovium in animal models and in the joint fluid in human RA, including vascular endothelial growth factor (VEGF) [19-23] and basic fibroblast growth factor (FGF-2) [24,25]. In preclinical animal models, pharmacological inhibition of angiogenesis with specific inhibitors to VEGF and FGF-2 results in attenuation of joint swelling and destruction typically associated with RA disease progression [26-29]. Further, a variety of other angiogenesis inhibitors (thalidomide, taxol, angiostatin, endostatin positively impact preclinical models of RA [30-35] suggesting a critical contribution of these factors to disease pathology. However, to date these promising preclinical results have not been translated to positive clinical outcomes.

The antiangiogenic activity of 2ME2 has been demonstrated *in vivo *in a number of systems including the corneal micropocket and chick chorioallantoic assays [5]. In addition, administration of 2ME2 leads to a reduction of tumor vasculature in a variety of tumor models [5,6]. *In vitro *studies have demonstrated that 2ME2 inhibits endothelial cell proliferation, migration and tube formation. 2ME2 treatment also inhibits microvessel branching from rat aortic rings growing in three-dimensional collagen gels. We assessed the impact of 2ME2 treatment on angiogenesis in a murine collagen-induced model of RA. Oral treatment with 2ME2 (100 mg/kg/day) completely abrogated staining of articular regions for vWF as compared to control-treated mice. In addition, oral treatment with 2ME2 (100 mg/kg/day) inhibited the expression of both VEGF and FGF-2 in the joint space as compared to control-treated mice. Collectively, these data suggest that 2ME2 inhibits the neovascular response elicited in this collagen antibody-induced model of RA and suggests that at least a part of the antiarthritic mechanism involves the down modulation of the proangiogenic cytokines, VEGF and FGF-2.

In addition to neovascularization, another early event in preclinical models of RA is the mobilization and infiltration of leukocytes into the synovium and articular fluid as well as intense inflammation. Following treatment with 2ME2 in this model, we observed a marked attenuation of synovial leukocyte infiltrate histologically. To further explore this 2ME2-mediated effect, we looked at the expression of inflammatory cytokines in resected joints. The inflammatory cytokines, TNF-α, IL-1β, IL-6 and IL-17 are all regulatory factors responsible for the initiation, perpetuation and destructive capacity of the rheumatoid arthritis synovium and all four have been implicated in recruitment of inflammatory cells to the joint [36]. In mice treated daily with 2ME2, the expression levels of TNF-α, IL-1β, IL-6 and IL-17 were suppressed early (study day 5) and remained suppressed when assessed on study day 14. The impact of 2ME2 treatment on cytokine expression and its relationship to the antiarthritic activity continues to be investigated.

The progressive destruction of the structural components of the joints involving the articular cartilage, the bone at the joint margins, as well as periarticular and subchondral bone, is another hallmark in the progression of RA. The impact of 2ME2 treatment on cartilage and bone destruction in this murine CAIA model was assessed by histomorphology and radiography. Oral treatment with 2ME2 demonstrated a dose-dependent inhibition of bone resorption, and ablated osteoclast activity and number when compared to control-treated mice as determined by TRAP staining. Parallel analyses also indicated the sparing of articular cartilage damage as measured by proteoglycan loss and maintenance of total cartilage thickness. As indicated above, treatment with 2ME2 in this model is associated with an inhibition in the expression of TNF-α, IL-1β, IL-6 and IL-17, VEGF, and FGF-2 in resected joints. These cytokines are abundant in inflamed synovium and produce marked disturbances of bone and cartilage remodeling leading to destruction of the extracellular matrices of these tissues [37,38]. In addition, it is documented that, FGF-2 directly accelerates osteoclast maturation and promotes bone resorption [39]. Hence, the impact on the expression of these inflammatory and angiogenesis factors by 2ME2 is likely be at least partially responsible for the striking attenuation and almost complete inhibition of tissue destruction observed in the mouse collagen antibody-induced model of arthritis.

Finally, we demonstrate that the development of pannus or granulation tissue that grows over and invades articular cartilage is also positively influenced by 2ME2. In this model, oral treatment with 2ME2 demonstrated a dose-dependent inhibition of pannus formation. This activity may occur as a result of the inhibitory effects of 2ME2 on angiogenesis and inflammation as noted above. However, it is not understood if additional described mechanisms of 2ME2 action (induction of apoptosis and inhibition of proliferation) may also be relevant for the observed antiarthritic activity [5,6]. 2ME2 targets actively proliferating cells and is not cytotoxic to quiescent cells. The antiproliferative activities of 2ME2 have been attributed to several mechanisms, including effects on superoxide dismutase enzymatic activity and microtubule structures [7-9]. Disruption of the microtubule cytoskeleton by 2ME2 results in the inhibition of expression and activity of the transcription factor, HIF-1α[10]. This results in a reduction in expression of the HIF-1α responsive gene, VEGF. Interestingly, in the context of RA, cytoplasmic and nuclear overexpression of HIF-1α is described in the synovial lining in rheumatoid arthritis patients offering another potential target for antiarthritic activity of 2ME2.

## Conclusion

2ME2 exerts pre-clinical antiarthritic activity in a mouse collagen antibody induced model of RA through multiple disease modifying actions: (1) inhibition of articular angiogenesis, (2) attenuation of inflammatory cell infiltration into the synovial compartment, (3) abrogation of soft tissue and bone lesions, and (4) arrest of pannus development. Whether these effects occur as the consequence of 2ME2 impacting a single pathway or multiple pathways continues to be investigated. Taken together, the disease-modifying activity of 2ME2 following oral administration in the absence of overt toxicity make 2ME2 an attractive and suitable therapeutic agent for treating rheumatoid arthritis and warrants clinical evaluation.

## Competing interests

The authors declare that they are all employees of EntreMed, Inc. and as such receive a salary from EntreMed, Inc. The corresponding author had full access to all of the data generated in the study and had final responsibility for the decision to submit for publication.

## Authors' contributions

SMP conceived of the study, participated in acquisition of the data, data analysis and interpretation, manuscript preparation, and approved the final manuscript. EJP, SJS, and EGM participated in data acquisition and data analysis and approved the final manuscript. CSF participated in data interpretation and approved the final manuscript. WEF participated in study design, participated in acquisition of the data and manuscript preparation, and approved the final manuscript.

## Pre-publication history

The pre-publication history for this paper can be accessed here:


